# Isolated Rapidly Progressive Interstitial Lung Disease Without Muscle or Skin Involvement in Idiopathic Inflammatory Myopathy: An Unusual Anti-Mi-2 Antibody Phenotype

**DOI:** 10.7759/cureus.89753

**Published:** 2025-08-10

**Authors:** Rajesh Kumar, Sushil Kumar, Ruthra Kumaran, Chandan Kumar, Kanchan K Kujur

**Affiliations:** 1 Internal Medicine and Rheumatological Services, All India Institute of Medical Sciences Deoghar, Deoghar, IND; 2 General Medicine, All India Institute of Medical Sciences Deoghar, Deoghar, IND; 3 Internal Medicine, All India Institute of Medical Sciences Deoghar, Deoghar, IND

**Keywords:** amyopathic dermatomyositis, anti-mi-2, idiopathic inflammatory myopathy, ild without myositis, rapidly progressive interstitial lung disease

## Abstract

Anti-Mi-2 antibodies are myositis-specific autoantibodies typically associated with cutaneous manifestations in idiopathic inflammatory myositis with favourable outcomes. Interstitial lung disease (ILD), particularly rapidly progressive ILD (RP-ILD), is rarely linked to anti-Mi-2 and even more infrequently presents without muscle or skin involvement.

We report a rare case of a 58-year-old male patient presenting with isolated RP-ILD without cutaneous or muscular manifestations. Laboratory evaluation revealed normal muscle enzymes and negative autoimmune markers, with myositis profile revealing isolated anti-Mi-2β antibody positivity, confirmed by enzyme-linked immunosorbent assay (ELISA) and immunoblot. Radiological features on high-resolution CT, coupled with the rapidity of progression, were suggestive of RP-ILD. Despite initial improvement with pulse corticosteroids and cyclophosphamide, the patient discontinued treatment and succumbed to the illness within two months.

This case highlights an extremely rare anti-Mi-2 phenotype presenting solely with RP-ILD. Although anti-Mi-2 is typically linked to benign clinical courses, this case underscores its potential association with severe pulmonary manifestations in the absence of classic dermatomyositis features.

Clinicians should be aware of atypical anti-Mi-2 presentations, including isolated RP-ILD. Early recognition and aggressive immunosuppressive therapy are critical to improving outcomes. Further research is essential to understand the underlying mechanisms and optimal management strategies in such presentations.

## Introduction

Idiopathic inflammatory myopathies (IIMs) are rare autoimmune disorders with a prevalence of approximately 2.4-33.8 per 100,000 population, characterized by chronic muscle inflammation and systemic involvement of skin and occasionally lungs [1]. Approximately 5%-10% of IIM cases manifest as ‘amyopathic’ or ‘clinically amyopathic dermatomyositis’ (ADM/CADM), presenting primarily with cutaneous but without muscle involvement. Interstitial lung disease (ILD), a life-threatening extra-muscular manifestation, occurs in 20%-86% of IIM cases. ILD most often coexists with or follows myositis but may precede it in rare instances. Myositis-specific antibodies (MSAs), such as anti-MDA5, Mi-2, TIF1γ, Jo-1, NXP2, and anti-aminoacyl-tRNA synthetase (ARS), and myositis-associated antibodies (MAAs), such as anti-Ro52, anti-PM/Scl, and anti-Ku, are pivotal in diagnosing, prognosticating, and managing IIM, even when muscle involvement is absent [1].

Anti-Mi-2 antibodies, in contrast, are usually linked to classic dermatomyositis with cutaneous involvement and rarely cause ILD and when they do, it is generally mild [2,3]. Rapidly progressive ILD (RP-ILD) is a rare, severe form of ILD characterized by rapid respiratory decline over three months, with worsening dyspnea, hypoxemia, and radiographic progression. RP-ILD is frequently associated with anti-MDA5 and anti-ARS antibodies and typically predicts a poor prognosis [4]. We present an extraordinary case of isolated anti-Mi-2-related RP-ILD occurring without muscle or skin manifestations, illustrating the diagnostic complexity and extending the clinical spectrum of IIM [5,6]. 

## Case presentation

A 58-year-old male truck driver presented with the onset of progressive dyspnea (modified Medical Research Council (mMRC) Dyspnea Scale increased from 1 to 4) and a nonproductive cough over three months. He reported no arthritis, fever, weight loss, muscle pain, proximal weakness, dysphagia, Raynaud’s phenomenon, cutaneous signs, sicca symptoms, or gastroesophageal reflux. There was also no history of occupational dust exposure, animal contact, medications, addiction, or comorbid conditions.

On examination, he exhibited no evidence of classic dermatomyositis skin findings, such as heliotrope rash, Gottron’s papules, or shawl sign. His respiratory rate was 36 breaths/min, and oxygen saturation was 80% on room air, requiring 4 L/min via nasal cannula upon hospital admission. Neurological assessment revealed normal strength (5/5) in all limbs. Lung auscultation noted scattered Velcro crackles. Cardiovascular and musculoskeletal examinations were unremarkable.

**Figure 1:**
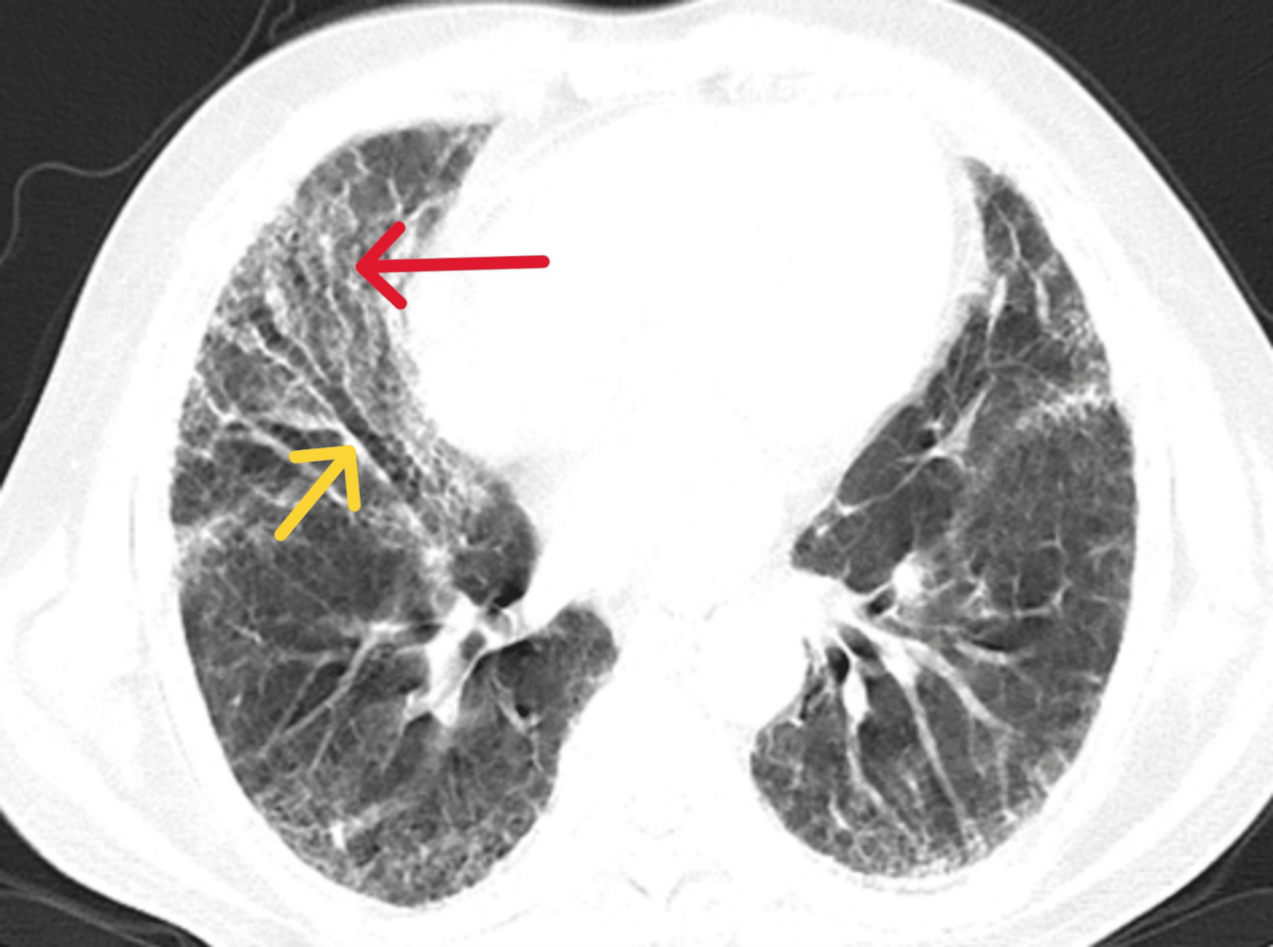
Bilateral diffuse reticular opacities and ground glass opacities (GGOs) (red arrowhead) with traction bronchiectasis (yellow arrowheads) and lung architectural distortion more involving in the lower lobes.

**Figure 2:**
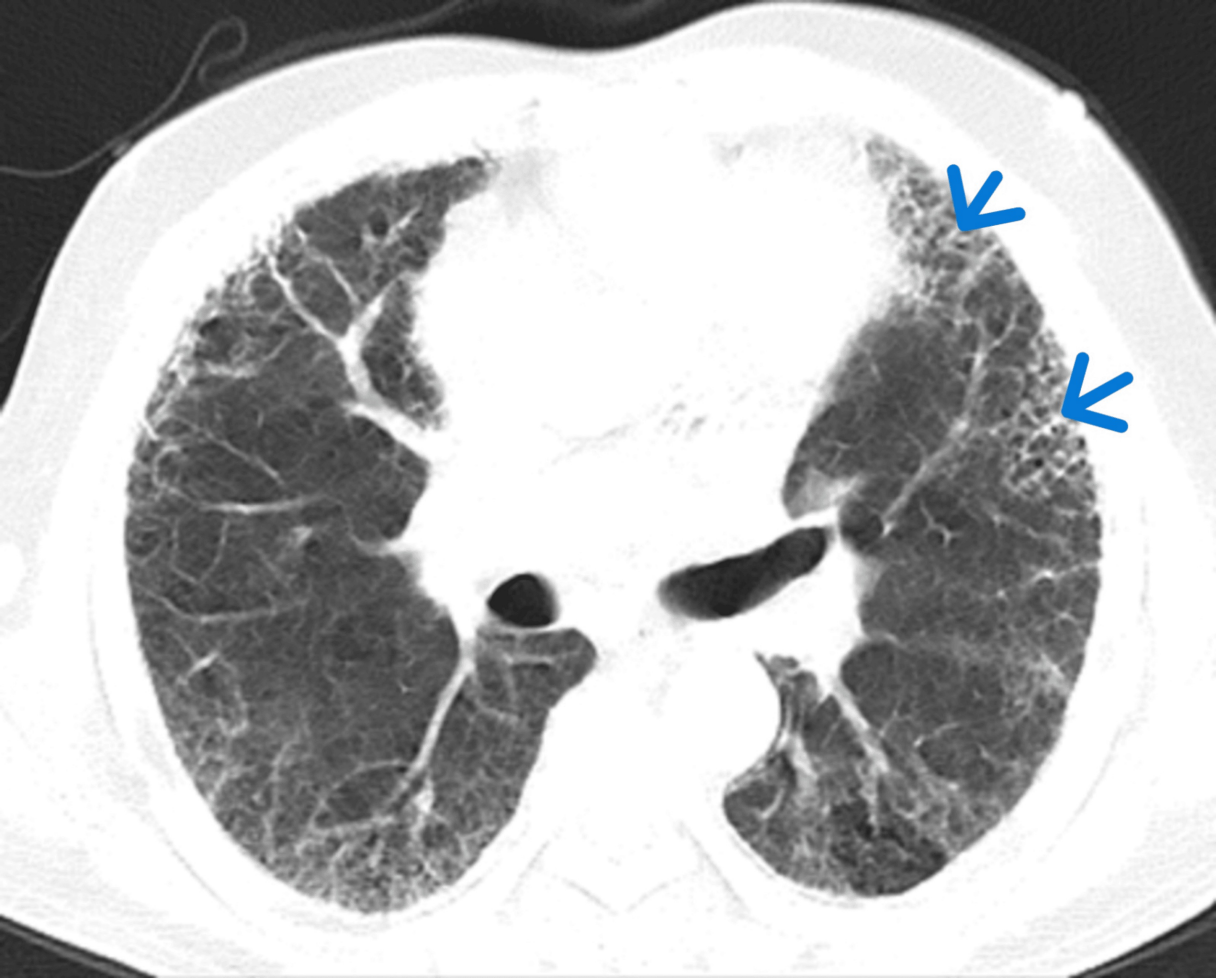
Bilateral diffuse reticulations and ground glass opacities (GGOs) with inter septal thickening in middle lobe (blue arrowheads).

**Figure 3:**
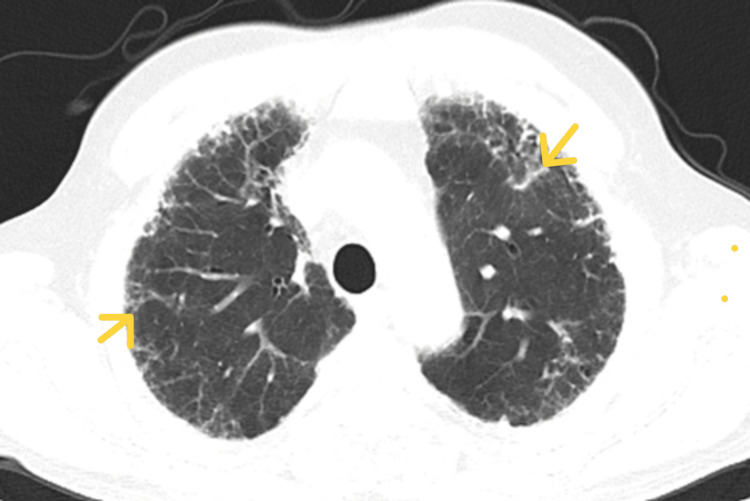
Mild subpleural reticulations in upper lobe (yellow arrowhead).

Pulmonary function testing could not be done due to poor respiratory effort. There was no radiological evidence of malignancy. High-resolution CT findings were consistent with fibrotic nonspecific interstitial pneumonia (NSIP) affecting all lobes, predominantly in the lower lobes (Figures 1-3). 

Laboratory investigations revealed normal complete blood count (CBC), renal, liver, thyroid, and glucose profiles; muscle enzymes (creatine phosphokinase (CPK), aldolase, serum glutamic-oxaloacetic transaminase (SGOT)) remained within normal limits (Table 1).

**Table 1:** Summary of Relevant Investigations with Patient Values and Reference Ranges Hb: hemoglobin, TLC: total leukocyte count, ESR: erythrocyte sedimentation rate, CRP: C-reactive protein, TSH: thyroid-stimulating hormone, SGOT: serum glutamic-oxaloacetic transaminase , SGPT: serum glutamic-pyruvic transaminase , ALP: alkaline phosphatase, RA: rheumatoid factor, Anti-CCP: Anti-cyclic citrullinated peptide, ANA IFA: antinuclear antibody by indirect immunofluorescence assay, ANA profile: antinuclear antibody Profile, NT-proBNP: N-terminal pro B-type natriuretic peptide, CPK-MM: creatine phosphokinase – muscle isoform, USG: ultrasonography.

Investigation	Patient Value	Normal Range
Hb	12 g/dl	13-17 g/dL
TLC	7400/µL	4,000-11,000 /µL
Platelets	1,30,000/µL	150,000-450,000 /µL
ESR	60 mm/h	0-20 mm/hr (male)
CRP	22 mg/L	0-10mg/L
FBS	98mg/dl	70-100mg/dL
HbA1c	4.90%	4 - 5.6%
TSH	3.4 µIU/mL	0.27-4.2µIU/mL
Urea	36 mg/dl	13-43 mg/dL
Creatinine	0.7 mg/dl	0.7-1.3 mg/dL
Urine routine	no protein/RBC	
Bilirubin (T)	0.8 mg/dL	0.3-1.2 mg/dL
Bilirubin (C)	0.6 mg/dL	0.0-0.4 mg/dL
SGOT	48 U/L	<40 U/L
SGPT	28 U/L	<40 U/L
ALP	131 U/L	44-147 U/L
Protein	6.8 g/dL	6.0-8.3 g/dL
Albumin	2.6 g/dL	3.5-5.0 g/dL
Globulin	2.7 g/dL	2.0-3.5 g/dL
RA	<8 IU/ml	<14 IU/ml
Anti-CCP	<10 U/ml	<17 U/ml
ANA IFA	Negative	
ANA Profile	No antibody present	
NT-proBNP	100 pg/mL	125 pg/mL
CPK-MM	98 U/L	10-120 U/L
Aldolase	5 U/L	1.0 to 7.5 U/L
USG abdomen	Normal study	
Serum Procalcitonin	0.05 ng/mL	0.1 ng/mL

Arterial blood gas analysis indicated type 1 respiratory failure. Viral serologies were negative; sputum Gram stain, acid-fast bacilli (AFB) stain, and cartridge-based nucleic acid amplification test (CBNAAT) were negative. Autoimmune panels (RF, anti-CCP, ANA) were negative (Table 1). The myositis-specific panel revealed isolated anti-Mi-2β antibody positivity via enzyme-linked immunosorbent assay (ELISA) and immunoblot (Table 2).

**Table 2:** Myositis profile on immunoblot assay showing only anti-Mi 2 β-antibodies.

Antigen	Class
Mi-2alpha	o
Mi-2beta	++
TIF1 gamma	o
MDA5	o
NXP2	o
SAE1	o
Ku	o
PM-Scl100	o
PM-Scl75	o
Jo-1	o
SRP	o
PL-7	o
PL-12	o
EJ	o
OJ	o
Ro52	o

Muscle biopsy was not performed as the patient did not give consent. Mini-bronchoalveolar lavage showed no evidence of viral, bacterial, or fungal infection. Echocardiography and NT-proBNP (100 pg/mL) were normal.

The patient received three days of pulse-dose corticosteroids followed by oral steroids and cyclophosphamide. He demonstrated initial improvement and was discharged, but discontinued treatment and died two months later.

## Discussion

Isolated anti-Mi-2 positivity leading to RP-ILD without dermatomyositis or polymyositis manifestations are exceedingly rare, with very limited published case reports and no large cohorts [7]. In our patient, muscle power and muscle enzymes were normal, and there was neither any history of skin rash nor any cutaneous findings on examination. It has been mentioned in literature that if only anti-Mi-2 is found in RP-ILD-associated amyopathic IIM, then the myositis profile should be cross-verified, as it is mostly associated with anti-MDA5 or ARS. In our case, it was cross-verified, and in both, only anti-Mi-2 was found. The prognosis of anti-Mi-2-associated ILD is generally more favourable than that associated with anti-MDA5, ARS antibodies [2,8,9]. However, the rapid progression observed in this case underscores the variable nature of anti-Mi-2-related diseases and the need for vigilant monitoring, timely intervention, and further research.

RP-ILD is a rare and severe complication often associated with IIMs, particularly DM [5,6]. Its occurrence in patients with anti-Mi-2 antibodies is exceedingly rare, broadening the clinical spectrum of these autoantibodies in IIMs [3,5]. In this case, RP-ILD was considered, as there was a rapid worsening of respiratory symptoms within three months, and the lower lobes were more involved compared to the other lobes.

A meta-analysis of 18 studies involving 6,058 DM/PM (dermatomyositis/polymyositis) patients found an RP-ILD prevalence of approximately 8.9% [6]. Anti-Mi-2 antibodies are MSAs, directed against components of the nucleosome remodelling deacetylase (NuRD) complex and exist in two subtypes, Mi-2α and Mi-2β, which are generally indistinguishable. They are found in approximately 4%-18% of DM cases, and are 98%-100% specific with poor sensitivity [2,3,9]. Anti-Mi-2 antibodies are primarily associated with DM and are characterized by hallmark skin manifestations, such as the V-sign, Shawl sign, and Gottron’s papules, alongside a favorable prognosis and responsiveness to immunosuppressive therapy [2,9]. This atypical presentation poses significant diagnostic and therapeutic challenges, as seen in this case.

The pathogenesis of ILD associated with anti-Mi-2 antibodies is not well understood. Immune-mediated inflammation targeting both muscle and pulmonary tissues, potentially influenced by genetic predisposition and environmental triggers, is believed to play a central role [7]. Persistent inflammation within the alveolar and interstitial spaces can lead to fibrosis and architectural distortion of the lungs, contributing to the rapid clinical deterioration seen in RP-ILD.

The management of RP-ILD requires aggressive immunosuppressive therapy. High-dose corticosteroids form the cornerstone of treatment, often supplemented with additional agents such as cyclophosphamide, mycophenolate mofetil, or calcineurin inhibitors [7,8].

## Conclusions

This case highlights an exceptionally rare manifestation of anti-Mi-2-positive IIM presenting as isolated RP-ILD without skin or muscle involvement. It emphasizes the need for clinician awareness of a multidisciplinary approach in cases of atypical anti-Mi-2 phenotypes and the importance of early, aggressive therapy. Future research is necessary to uncover the mechanisms, biomarkers, and optimal management of this unusual presentation.
